# Revolutionizing multi‐omics analysis with artificial intelligence and data processing

**DOI:** 10.1002/qub2.70002

**Published:** 2025-04-07

**Authors:** Ali Yetgin

**Affiliations:** ^1^ Research and Development Center Toros Agri Industry and Trade Co. Inc. Mersin Turkey; ^2^ Department of Biotechnology Institute of Nature and Applied Sciences Cukurova University Adana Turkey

**Keywords:** artificial intelligence, data processing, deep learning, machine learning, multi‐omics, neural networks

## Abstract

Our understanding of intricate biological systems has been completely transformed by the development of multi‐omics approaches, which entail the simultaneous study of several different molecular data types. However, there are many obstacles to overcome when analyzing multi‐omics data, including the requirement for sophisticated data processing and analysis tools. The integration of multi‐omics research with artificial intelligence (AI) has the potential to fundamentally alter our understanding of biological systems. AI has emerged as an effective tool for evaluating complicated data sets. The application of AI and data processing techniques in multi‐omics analysis is explored in this study. The present study articulates the diverse categories of information generated by multi‐omics methodologies and the intricacies involved in managing and merging these datasets. Additionally, it looks at the various AI techniques—such as machine learning, deep learning, and neural networks—that have been created for multi‐omics analysis. The assessment comes to the conclusion that multi‐omics analysis has a lot of potential to change with the integration of AI and data processing techniques. AI can speed up the discovery of new biomarkers and therapeutic targets as well as the advancement of personalized medicine strategies by enabling the integration and analysis of massive and complicated data sets. The necessity for high‐quality data sets and the creation of useful algorithms and models are some of the difficulties that come with using AI in multi‐omics study. In order to fully exploit the promise of AI in multi‐omics analysis, more study in this area is required.

## 1 INTRODUCTION

A biological research strategy known as “multi‐omics analysis” involves the concurrent examination of various kinds of molecular data from a biological sample. Genomic, transcriptomic, proteomic, metabolomic, and other “omics” technologies can produce these data types. With the use of multi‐omics analysis, researchers can gain a more complete understanding of biological systems and the intricate relationships between various molecules and metabolic pathways. Multi‐omics ana‐lysis helps generate personalized medical strategies, find novel biomarkers, and shed light on previously unknown illness caused by integrating a variety of data types [1]. Large and complicated data sets are produced during multi‐omics analysis, necessitating the use of sophisticated computational and statistical techniques. Integrating metabolomics data, for instance, can assist in identifying metabolic pathways linked to certain diseases, whereas integrating genomes and proteomics data can assist in identifying post‐transcriptional alterations that affect protein function.

The domain of multi‐omics research has transitioned from discrete single‐omics investigations to a holistic, integrative methodology, propelled by innovations in high‐throughput technologies and bioinformatics. Initial inquiries predominantly centered on genomics and transcriptomics, elucidating the genetic underpinnings of diseases and their associated regulatory frameworks. Nonetheless, these methodologies frequently proved inadequate in elucidating phenotypic intricacies, as they overlooked the contributions from proteomics, metabolomics, and epigenomics. The advent of mass spectrometry, next‐generation sequencing, and sophisticated data integration in the early 2000s facilitated researchers in examining the interactions among various molecular strata, signifying a paradigm shift towards systems biology [2]. This integrative methodology gained heightened prominence alongside the emergence of precision medicine initiatives; wherein multi‐omics elucidated patient‐specific molecular profiles. Currently, the amalgamation of multi‐omics data not only augments our comprehension of biological systems but also expedites drug discovery, disease diagnosis, and the formulation of personalized therapeutic strategies, thereby cementing its status as a fundamental pillar of contemporary biomedical research.

**FIGURE 1:**
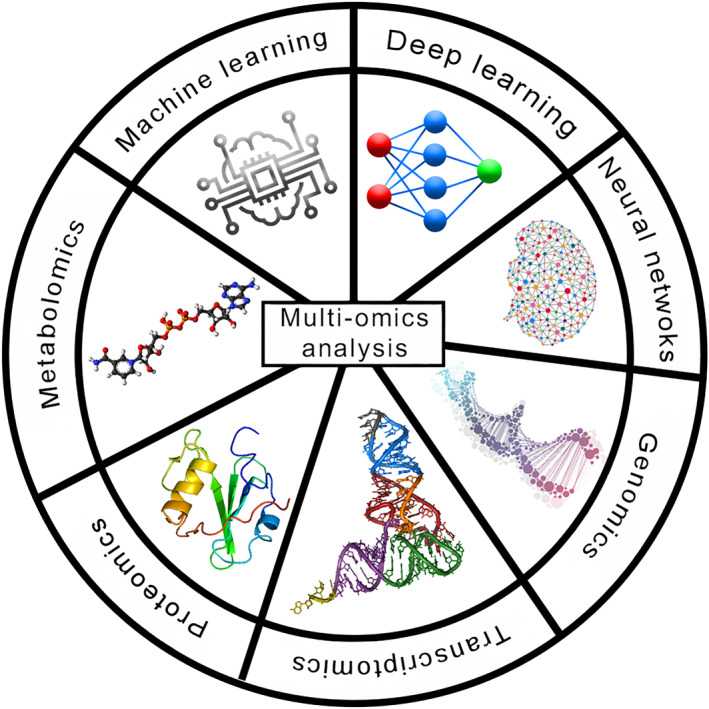
Integration of artificial intelligence with multi‐omics analysis.

Figure 1 demonstrates how biological research may be revolutionized by the combination of artificial intelligence (AI) and multi‐omics technologies, resulting in more complete insights and improvements in disciplines including systems biology, biotechnology, and personalized medicine. The importance of multi‐omics analysis is rising in areas including cancer research, pharmaceutical development, and precision medicine. Our comprehension of intricate biological systems and the development of fresh insights into the underlying molecular causes of disease are both being advanced by the integration of various omics data types [3, 4]. However, the integration and analysis of multi‐omics data sets can be difficult and call for specialized knowledge across a number of fields, cutting‐edge computational resources, and the creation of novel analytical techniques. Multi‐omics analysis is a formidable strategy that has the potential to revolutionize the way we understand biological systems as well as how novel cures and treatments are created.

The advent of sophisticated AI instruments, exemplified by ChatGPT, has precipitated a profound transformation in the realms of bioinformatics and multi‐omics inquiry. ChatGPT represents a substantial language processing model proficient in the generation of contextually pertinent textual content, facilitating navigation through intricate analytical endeavors, and aiding in the generation of programming code [5]. This multifaceted capability has the potential to optimize workflows related to omics data analysis by providing instantaneous support for scripting, debugging, and addressing prevalent computational obstacles. For example, researchers frequently encounter challenges in the integration of heterogeneous omics data types owing to discrepancies in formats and preprocessing specifications [6]. ChatGPT can mitigate such difficulties by delivering customized illustrations of data normalization, imputation strategies, or algorithmic selection, thereby effectively diminishing the learning curve for novices and augmenting the productivity of seasoned practitioners. Its incorporation into the bioinformatics framework serves as a testament to the capacity of generative AI to democratize access to advanced analytical instruments and methodologies.

In addition to its capacity as a coding facilitator, ChatGPT also serves as an invaluable asset for the generation of hypotheses and the synthesis of knowledge [7]. Through the analysis and summarization of extensive volumes of scientific literature, it can discern emerging trends, generate concepts for omics investigations, or propose pertinent experimental frameworks. In the context of multi‐omics research, wherein various datasets encompassing genomics, proteomics, and metabolomics intersect, ChatGPT’s adeptness at managing large‐scale textual information ensures that researchers remain informed of the latest developments. Furthermore, its utility extends to the instruction of complex machine learning methodologies, encompassing neural network optimization and dimensionality reduction techniques specifically adapted for high‐dimensional omics data. As a readily accessible, interactive, and continuously advancing AI, ChatGPT complements conventional bioinformatics resources, thereby fostering innovation within integrative omics analysis and precision medicine [8].

Because of the complexity and size of the data involved, processing and analyzing multi‐omics data presents substantial problems (Table 1). To fully comprehend biological systems, different forms of biological data, such as genomics, transcriptomics, proteomics, metabolomics, and epigenomics, are combined to form multi‐omics data. The integration and interpretation of these various datasets, however, are challenging and call for specific methods. Data integration is among the biggest difficulties. Each omics dataset has unique properties, including various data formats, measurement techniques, and data quality [15]. It takes advanced techniques to properly align and normalize the data when integrating these databases. Large‐scale datasets from various sources must also be integrated, which presents technical difficulties regarding data storage, computing power, and data transfer.

**TABLE 1:** Challenges associated with multi‐omics data processing and analysis.

Challenge	Description	Tools/techniques	Applications	References
Data integration	Integration of multiple omics data types requires careful consideration of technical and biological variability, data normalization, and data imputation	Co‐inertia analysis, and joint and individual variation explained (JIVE)	Cancer research, drug development, and precision medicine	[9]
Data dimensionality	Multi‐omics data sets can be high‐dimensional, meaning that they include many variables that may be correlated or redundant, making it challenging to identify the most important features	Principal component analysis (PCA), *t*‐distributed stochastic neighbor embedding (*t*‐SNE)	Biomarker discovery, disease diagnosis	[10]
Statistical analysis	Multi‐omics data sets require advanced statistical techniques, such as machine learning and network analysis, to identify patterns and interactions within the data	Random forest, support vector machines (SVM)	Drug discovery, disease subtyping	[11]
Computational resources	Multi‐omics data sets can be computationally intensive and require significant computational resources and expertise to process and analyze	High‐performance computing, cloud computing, and parallel computing	Personalized medicine and patient stratification	[12]
Interpretation and visualization	Multi‐omics data sets can be difficult to interpret and visualize, requiring the development of new approaches to identify meaningful biological insights	Heatmaps, pathway analysis, and gene ontology (GO) analysis	Functional annotation, pathway analysis, and identification of biomarkers	[13]
Reproducibility	The large and complex nature of multi‐omics data sets can make it challenging to reproduce and validate results, highlighting the need for transparency and standardized approaches	Workflow management systems, reproducible research	Clinical trials and drug development	[14]

## 2 MULTI‐OMICS DATA TYPES

Due to the size and complexity of the data produced by various omics technologies, AI and data processing are essential elements in multi‐omics analysis [16]. Multiple omics technologies, including genomics, transcriptomics, proteomics, and metabolomics, can contribute a significant amount of data to multi‐omics data sets. Researchers can handle and analyze these massive, diverse data sets with the aid of AI and data processing techniques. Automating and streamlining data processing and analysis workflows is one of AI’s key advantages in multi‐omics study [17]. For instance, patterns and linkages within multi‐omics data sets can be found using machine learning algorithms, which are difficult or impossible to find using conventional statistical techniques. These algorithms can also assist researchers in reducing the dimensionality of multi‐omics data sets, enabling them to prioritize future analysis and pinpoint the most crucial aspects.

There were more efficiently integrated and visualized multi‐omics data sets with the use of AI and data processing techniques in addition to automating and streamlining procedures. For instance, AI‐based data integration techniques can assist researchers in finding connections between various omics data types, allowing them to construct more thorough models of biological systems [18, 19]. Similar to how complicated multi‐omics data sets can be visualized more easily and effectively, enhanced data visualization techniques can aid in the discovery of important biological insights. AI and data processing techniques will definitely play an increasingly significant role in aiding new discoveries and enhancing human health as multi‐omics analysis continues to develop.

**FIGURE 2:**
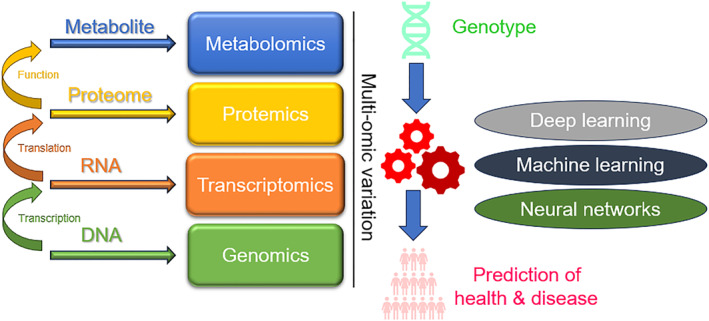
Artificial intelligence support of multi‐omic variation for prediction of health and disease.

AI plays a crucial role in leveraging multi‐omic variation for predicting health and disease outcomes (Figure 2). Multi‐omics refers to the integration of data from various biological sources, such as genomics, transcriptomics, epigenomics, proteomics, meta‐bolomics, and other omics disciplines. Combining information from these diverse data sets allows researchers and clinicians to gain a more comprehensive understanding of the molecular mechanisms underlying health and disease. Finding biomarkers and possible therapeutic targets for diverse diseases is another significant benefit of AI and data processing in multi‐omics study [20, 21]. These advanced methodologies often rely on feature importance analysis in conjunction with model performance metrics to pinpoint the most relevant biomarkers and potential therapeutic targets. Subsequently, these findings can undergo experimental investigations to further validate their significance. These are important for finding potential toxicity problems and side effects of medications and other therapeutic agents with the aid of AI and data processing [22, 23]. By analyzing multi‐omics data from various tissues and cell types, it is possible to identify potential off‐target effects of medications and assess their impact on overall health. This information can be used to guide the development of new drugs, ultimately improving patient outcomes.

Researchers can also get beyond some of the drawbacks of conventional single‐omics analysis with the aid of AI and data processing. For instance, researchers found posttranscriptional and posttranslational alterations that single‐omics analysis would miss by combining data from genomes, transcriptomics, and proteomics [24]. The mechanisms behind many biological processes and disorders may become clearer as a result. For the analysis and interpretation of the enormous amount of data produced by multi‐omics technologies, AI and data processing are crucial. These techniques make it easier to find biomarkers, pharmacological targets, and potential negative effects of medications and other therapeutic agents. Additionally, they can aid in overcoming the restrictions of conventional single‐omics analysis and offer a more thorough and precise picture of biological systems.

In multi‐omics research, it is important to provide a detailed explanation of the specific instrumental setups used and the types of data they generate. This approach enhances our understanding of the complexities involved in data acquisition and its potential implications. This exploration should encompass various data types, such as read counts and mass concentration, among others, which are crucial in the multi‐omics context [25]. Understanding the differences in instrumental setups is essential, as each technology or method possesses its strengths and weaknesses. For instance, certain setups may excel in generating high read counts, making them ideal for analyzing gene expression patterns. On the other hand, other methodologies may be more adept at measuring mass concentration accurately, which is particularly relevant in metabolomics or proteomics studies.

### 2.1 Genomics

It is significant to understanding an organism’s whole DNA sequence, including all of its genes and non‐coding regions. This sort of data includes analyzing DNA’s sequence, structure, and function as well as locating genetic variants and determining how they relate to specific traits and illnesses. DNA sequencing and genotyping are two of the main technologies used in genomics. While genotyping entails the detection of genetic variations, such as single nucleotide polymorphisms (SNPs), in a particular region of the genome, DNA sequencing involves determining the order of nucleotides in a DNA molecule [26].

Applications for genomic data include finding disease‐causing mutations, forecasting treatment responses, and population genetics research. Genomic information has been utilized, for instance, to pinpoint cancer cell mutations that promote tumor growth and to create tailored treatments that specifically target these mutations [27]. Additionally, it has been used to pinpoint genetic variations linked to drug response, paving the way for personalized medical treatments that take a person’s genetic profile into account. The crucial multi‐omics data type of genomics offers a plethora of knowledge regarding the genetic make‐up of an organism and its possible effects on health and disease.

For a more complete knowledge of biological systems, genomic data is frequently combined with other multi‐omics data types, such as transcriptomics and proteomics. For instance, proteomics data can be used to determine which proteins are being generated from those RNAs, while transcriptomics data can be used to determine which genes are actively being transcribed into RNA. Researchers can build a more thorough understanding of the molecular mechanisms underpinning numerous biological activities by merging these many sorts of data. Additionally, there are numerous ways to analyze genomics data, including variant calling, GWAS, and gene expression analysis [28]. Finding genetic differences in a sample, such as SNPs and copy number variations, is known as variant calling [29]. In GWAS, genetic variations that are linked to a particular trait or disease in a population are found. Measurement of the quantities of RNA transcripts generated from genes in a sample is a component of gene expression analysis.

The sheer amount of genomics data is one of the biggest problems. Terabytes of data can be produced by DNA sequencing, and processing and evaluating these data call for specialized computational resources and knowledge [30, 31]. However, recent years have seen an increase in the availability and affordability of genomics data due to developments in sequencing technology and data processing algorithms. The critical multi‐omics data type of genomics offers crucial insights into the genetic make‐up of an organism and its possible effects on health and disease. It can result in significant discoveries in a variety of domains, including personalized medicine, drug development, and evolutionary biology.

### 2.2 Transcriptomics

The study of an organism’s entire set of RNA transcripts, including messenger RNA and non‐coding RNA, is known as transcriptomics. This data type can offer insights into gene regulation, signaling networks, and physiological processes by revealing which genes are actively translated into RNA and at what quantities. RNA sequencing (RNA‐seq), which entails the sequencing of RNA transcripts and measurement of their expression levels, is the main technology used in transcriptomics [32]. Insights into the molecular mechanisms behind diverse biological processes can be gained from RNA‐seq data, which can be used to detect differentially expressed genes between various samples or situations.

Applications for transcriptomics data include discovering biomarkers for illness diagnosis and monitoring, locating therapeutic targets and predicting medication responses, and tracking the dynamics of gene expression during development and disease progression. For instance, gene expression signatures linked with particular cancer subtypes have been identified using transcriptomics data, and tailored medicines have been developed that particularly target these subtypes [33]. The complexity of transcriptomics data is one of its difficulties. Millions of reads per sample can be generated by RNA‐seq data, which necessitate specialized computer infrastructure and knowledge to process and analyze [34]. Additionally, sample preparation and RNA degradation are two additional sources of technical and biological variation that can have an impact on the accuracy and reproducibility of the results when applied to transcriptomics data. A potent multi‐omics data type called transcriptomics offers significant insights into the control and expression of genes [35]. In numerous disciplines, including personalized medicine, drug development, and developmental biology, its investigation may result in significant discoveries.

To provide a more thorough understanding of the biological processes, transcriptomics is frequently employed in conjunction with other multi‐omics data types, such as genomics and proteomics. For instance, transcriptomics data can be used to pinpoint the genes that are expressed differently under certain circumstances or disease states, and this knowledge can be paired with genomics data to pinpoint the genetic variants that are linked to these variations [36]. Similarly, proteomics information can be integrated with transcriptomics information to pinpoint the genes that are responsible for changes in protein expression or post‐translational modifications (PTMs) in response to certain stimuli. Transcriptomics has a significant role to play in systems biology, which uses quantitative data from several omics levels to simulate complex biologi‐cal systems [37]. Gene regulatory networks, which describe the relationships between genes and the proteins that control their expression, can be built using transcriptomics data. These networks can be used to discover important regulatory nodes that regulate the behavior of the entire network as well as to forecast how changes in one gene or protein will affect the expression of other genes.

Utilizing cutting‐edge data analysis methods and computational tools are crucial for making the most of transcriptomics data. In large‐scale transcriptomics datasets, patterns and relationships can be found, and AI and machine learning algorithms can be used to predict the functions of uncharacterized genes. Additionally, interactive, user‐friendly representations of transcriptomics data can be made using data visualization tools, which makes it simpler for researchers to explore and interpret their findings [38]. Transcriptomics is a significant multi‐omics data type that is essential to our comprehension of the regulation and expression of genes. It will continue to lead to significant discoveries in a variety of domains, from fundamental research to personalized medicine, thanks to its integration with other omics data types and cutting‐edge computational methods.

### 2.3 Proteomics

The study of all the proteins that are expressed by a cell, tissue, or organism is known as proteomics. It is a significant type of multi‐omics data that can shed light on the regulatory and functional networks that control cellular processes. Several methods, such as mass spectrometry, protein microarrays, and high‐throughput sequencing, can be used to produce proteomics data [39, 40, 41]. Finding biomarkers for disease diagnosis and prognosis is one of the primary uses of proteomics. Researchers can find proteins that are differently expressed, post‐translated, or mislocalized by studying the proteomes of patients with various disorders or disease stages. Then, these proteins can be utilized as indicators for diagnosis or prognosis or as potential drug targets.

Protein–DNA interactions, protein–small molecule interactions, and protein–protein interactions can all be studied using proteomics data. Researchers can pinpoint important signaling channels and regulatory networks that control biological activities by mapping these relationships. Proteomics data, for instance, can be used to map the downstream targets of a particular transcription factor (TF) and to identify the proteins that interact with it. In the discipline of structural biology, which seeks to comprehend the three‐dimensional structures of proteins and how they interact with other molecules, proteomics has another significant use. Protein complexes can be identified using proteomics data, and their structures can be examined using methods such as cryo‐electron microscopy or X‐ray crystallography [42]. Neural networks have significantly improved structural biology, especially in the realm of protein folding. Their ability to predict protein structures accurately and generate novel ones opens up new avenues for understanding biological processes, designing drugs, and advancing biotechnological applications.

Proteomics data demands sophisticated data processing methods and computational tools, just such as transcriptomics data does. Large‐scale proteomics datasets can be analyzed for patterns and correlations, and uncharacterized protein activities can be predicted using machine learning methods and AI. Proteomics is a crucial multi‐omics data type that is essential to our comprehension of how proteins function and are regulated [43]. The study and analysis of proteins are changing thank to a number of new emerging technologies and techniques in the fast developing discipline of proteomics. For instance, single‐cell proteomics is a recent strategy that enables researchers to examine the proteome of a single cell as opposed to examining a tissue or population of cells. This may shed light on cell‐to‐cell variation and facilitate the identification of fresh biomarkers or therapeutic targets. The investigation of PTMs is another newly developing field of proteomics [44]. PTMs are chemical alterations that take place after a protein is produced and have the potential to impact the protein’s structure, stability, and functionality. PTMs can be recognized, quantified, and their functional involvement in biological processes studied using proteomics techniques such as protein microarrays and mass spectrometry.

### 2.4 Metabolomics

The study of metabolites, or small molecules, which are found in biological fluids, tissues, and cells, is known as metabolomics. The results of cellular processes, or metabolites, can offer information about the functioning of cells as well as the effects of outside variables including nutrition, exercise, and exposure to the environment. Using mass spectrometry, nuclear magnetic resonance, or other analytical techniques, metabolites are often identified and quantified in metabolomics procedures [45]. These methods can produce a lot of data, which are then analyzed and combined with other omics data types using bioinformatics tools.

Finding biomarkers is one of the main uses of metabolomics. Changes in metabolite levels can be used to diagnose or track the development of an illness. Metabolites can act as disease markers. Metabolomics, for instance, has been applied to find biomarkers for different malignancies, diabetes, and cardiovascular disease [46]. The development of new drugs is a further use for metabolomics. A drug’s efficacy and safety can be tracked using metabolomics, which can also be used to discover the metabolic pathways that the medicine affects. Metabolomics can also be utilized to improve drug distribution and dose and discover new therapeutic targets.

One of the main issues is the diversity and unpredictability of the metabolome, which can make it difficult to identify and quantify metabolites. Additionally needed are standardized procedures, data analysis workflows, and more extensive data integration and exchange. Metabolomics, a significant data type in multi‐omics analysis, can be integrated with other omics data types to provide a more comprehensive understanding of biological systems and disease [37]. Metabolomics data can also be useful for tailored treatment and nutrition. By analyzing a person’s metabolome, which represents the complete set of metabolites present in their biological system, it becomes possible to identify their unique metabolic profile and customize interventions, such as pharmaceutical therapies or dietary adjustments, to suit their specific requirements.

In addition to applications in health and disease, metabolomics also has applications in toxicity monitoring and environmental monitoring. It is possible to utilize metabolomics to monitor long‐term changes in the environment and to ascertain how toxins and other environmental elements influence organisms [47]. There are some challenges associated with meta‐bolomics include the need for high‐quality samples, the significant variance in metabolite levels between individuals and populations. Additionally, there is a need for improved metabolite annotation and identification because many of them have not yet been identified or characterized.

### 2.5 Combination of multiomics data

The amalgamation of genomics and transcriptomics data facilitates a thorough comprehension of the molecular mechanisms that govern gene regulation and expression. Genomic data, comprising DNA sequences and genetic polymorphisms, can be correlated with transcriptomic data to investigate the impact of genomic variations on RNA expression profiles. Methodologies such as expression quantitative trait loci mapping establish connections between specific genetic polymorphisms and variations in gene expression [48]. Furthermore, techniques such as weighted gene co‐expression network analysis empower researchers to discern gene modules and regulatory networks that are conserved across various datasets [49]. By integrating single‐cell RNA sequencing (scRNA‐seq) with whole‐genome sequencing, researchers can examine the ramifications of genetic mutations on cellular heterogeneity and transcriptomic states, thereby facilitating advancements in precision medicine applications.

The integration of proteomics and metabolomics is imperative for elucidating biochemical pathways and metabolic networks. Methodologies such as joint pathway enrichment analysis enable the concurrent mapping of protein functionalities and metabolic flux dynamics. Correlation‐centric methodologies, including sparse canonical correlation analysis, can discern significant associations between proteins and metabolites, thereby facilitating the construction of metabolic and signaling pathways [50]. Datasets derived from mass spectrometry‐based proteomics and meta‐bolomics are frequently amalgamated through software solutions, such as Cytoscape, which offer visualization frameworks conducive to network‐based analyses. This integration is pivotal for the identification of biomarkers and the elucidation of PTMs that influence metabolic processes, thereby providing valuable insights into disease progression and potential therapeutic targets.

The synthesis of varied omics data types, including genomics, transcriptomics, proteomics, and meta‐bolomics, necessitates advanced computational frameworks. Multi‐omics integration typically employs machine learning methodologies, such as deep learning architectures or Bayesian network inference, to capture intricate relationships across datasets. Platforms such as multi‐omics factor analysis and mixOmics offer robust solutions for the integration and analysis of multi‐omics data, enabling researchers to uncover shared patterns and latent factors [51]. An additional emerging methodology involves graph‐based models, wherein nodes signify omics features, and edges delineate associations across omics layers. These frameworks are essential for constructing comprehensive models of biological systems, facilitating the discovery of regulatory hierarchies and cross‐omics biomarkers that are critical for systems biology and personalized medicine.

Proteomics is increasingly being combined with other omics data types, such as genomics and metabolomics. In order to find novel peptides and splice variants as well as to enhance the annotation of genomic sequences, one method is known as proteogenomics. Proteomics has made tremendous strides, however there are still a number of issues that need to be resolved [52, 53]. The complexity and dynamic range of the proteome, which can make it challenging to recognize and measure low‐abundance proteins, is one of the major problems. Standardized methods, workflows for data analysis, as well as increased data exchange and integration, are also required. Proteomics is a fundamental data type in multi‐omics analysis, and it will be crucial for expanding our understanding of biological systems and creating new treatments for human disease if it is developed further and integrated with other omics data types.

## 3 AI AND DATA PROCESSING TOOLS

Machine learning (ML) is a type of AI that employs algorithms to automatically find patterns in data and makes predictions or decisions on those patterns. Machine learning can be used in multi‐omics analysis to examine vast, complicated datasets and find significant properties (such as genes, proteins, or metabolites) that may be pertinent to a biological process [54]. Machine learning algorithms come in a variety of forms, such as supervised learning, unsupervised learning, and reinforcement learning. A model is trained under supervision using a labeled dataset in which each sample’s status for the outcome of interest (such as the presence or absence of a disease) is known. The outcome of additional samples can then be predicted using the model. Supervised learning can be applied in multi‐omics research to find biomarkers for illness diagnosis or therapy response prediction [55, 56]. Supervised learning relies on labeled data, enabling the model to make predictions with a clear target variable, but it may require a substantial amount of annotated data for training. On the other hand, unsupervised learning explores the inherent patterns and structures in unlabeled data, making it valuable for data exploration and clustering, but its predictions are more challenging to evaluate as it lacks explicit target labels [57]. Both approaches have distinct advantages and limitations, and their selection depends on the nature of the data and the specific objectives of the analysis.

Principal component analysis is a dimensionality reduction technique used to transform high‐dimensional data into a lower‐dimensional space while preserving the most significant patterns, making it valuable for data visualization and feature selection [58]. Random forest, an ensemble learning method, combines multiple decision trees to achieve robust predictions and is well‐suited for tasks such as classification and regression in diverse datasets. Hierarchical clustering, on the other hand, enables the grouping of data points into nested clusters based on similarity, aiding in the discovery of natural data structures. By detailing the key applications of these algorithms, readers can better understand their respective strengths and grasp their potential contributions to multi‐omics analysis with AI and data processing.

On the other hand, unsupervised learning entails training a model on an unlabeled dataset where the desired outcome is not known. As a result, the model can spot trends and classify samples according to how similar their omics profiles are. Unsupervised learning can be helpful in uncovering new disease pathways or processes as well as unique disease subtypes. A type of machine learning called reinforcement learning includes teaching a model to make decisions depending on input from its surroundings. Based on patient omics profiles and treatment outcomes, reinforcement learning in multi‐omics analysis can be used to determine the best treatment plans for a particular disease [55, 59, 60]. Performance validation is a critical aspect of ML model evaluation, ensuring the reliability and generalizability of the results. However, when dealing with high‐dimensional data, such as multi‐omics datasets, traditional validation techniques may encounter difficulties, as the curse of dimensionality can lead to overfitting and increased computational costs. Therefore, exploring specialized validation approaches tailored to handle the dimensionality challenges would be pertinent, valuable insights into effectively assessing the performance of machine learning algorithms in the context of multi‐omics analysis.

Deep learning, a subset of machine learning, is the process of learning and extracting intricate patterns and relationships from data using neural networks with numerous layers. Deep learning can be used in multi‐omics analysis to integrate and evaluate huge, complicated datasets that comprise several kinds of omics data [61]. For tasks, such as speech recognition, image recognition, and natural language processing, where the data is highly complicated and non‐linear, deep learning models are extremely helpful. These models, however, can also be used with omics data to identify significant features and trends that would be challenging or impossible to find using conventional statistical techniques.

Convolutional neural network (CNN), a prominent deep learning architecture for image recognition applications, is one deep learning architecture that is frequently utilized for multi‐omics research. By representing the omics data as a 1‐ or 2‐dimensional image, with the features represented as pixels or voxels, CNNs can also be used on the data. The recurrent neural network (RNN), which is frequently used for sequential data such as time‐series data or transcriptome data, is another well‐liked deep learning architecture [62]. Deep belief networks, autoencoders, and generative adversarial networks are just a few of the deep learning architectures that can be employed for multi‐omics research in addition to CNNs and RNNs. These models have the potential to shed fresh light on the underlying biology of many biological processes and reveal hidden patterns and correlations in omics data. Our understanding of intricate biological systems could be completely changed by the application of deep learning to multi‐omics research, which could also spur the creation of innovative diagnostic and treatment methods.

For multi‐omics analysis, deep learning has a number of advantages over conventional machine learning techniques. Thousands or even millions of variables might be present in multi‐omics datasets, making it difficult to examine them using conventional statistical techniques. By automatically extracting pertinent characteristics and lowering the dimensionality of the input, deep learning algorithms can handle this high‐dimensional data. Omics data are frequently complicated and non‐linear, hence they may be difficult to represent the relationships between variables using linear models [63]. Deep learning models may recognize non‐linear correlations between variables, resulting in more precise predictions and deeper understanding.

With little additional training, deep learning models can be used to transfer from one form of omics data to another. This makes it simpler to integrate many types of omics data since models created for one type of omics data may be applied to other types of data [64]. Large‐scale datasets can be used to train deep learning models, which enable the investigation of complex biological systems and the discovery of new biomarkers and therapeutic targets. Deep learning is an effective technique for multi‐omics research, and its use could revolutionize how we understand intricate biological functions and disorders.

Deep learning and neural networks are two closely intertwined concepts within the realm of AI and multi‐omics analysis. Specifically, deep learning is a refined subset of machine learning that exploits neural networks comprising multiple layers to extract intricate patterns and representations from data. In the context of multi‐omics analysis, deep learning techniques such as RNNs and CNNs have been devised to cope with the complexity of diverse molecular data types. These techniques can effectively learn hierarchical features and connections across different omics layers, thereby facilitating the integration and analysis of multi‐omics data. However, it is imperative to acknowledge challenges such as data quality and algorithm development when tapping into the potential of these AI‐driven approaches for multi‐omics research. Further advancements in this field are therefore warranted to fully harness the potential of deep learning and neural networks in revolutionizing our understanding of intricate biological processes [65].

Deep learning, a subset of machine learning, is a technique that entails learning and extracting intricate patterns and relationships from data using neural networks with multiple layers. The basic structure of neural networks enables them to process complex data representations through interconnected layers of nodes. Each layer progressively refines the learned features from the input data in subsequent layers. The optimization of neural networks requires adjusting the model’s parameters during training to minimize the variance between predicted and actual outputs [66]. This process, commonly achieved through back propagation, fine‐tunes the network’s performance and enhances its ability to generalize to new data. Nevertheless, the inherent complexity of deep learning models can present challenges in interpretability, making it challenging to comprehend the specific features influencing their decisions. Despite this, there are successful examples of translated neural networks in various fields, such as medical imaging and genomics, where deep learning models have demonstrated remarkable performance in diagnosing diseases and predicting treatment outcomes. The integration of neural networks in clinical practice has the potential to revolutionize personalized medicine, but it also requires continued research to ensure the robustness, reliability, and interpretability of the models for real‐world applications.

Multi‐omics analysis frequently employs neural networks, a type of AI and data processing technique. They are a type of computational model that can discover intricate patterns and connections within huge datasets, drawing inspiration from the structure and operation of the human brain. The ability of neural networks to learn from big and complicated datasets, which enables the study of high‐dimensional and heterogeneous omics data, is one of its key advantages. Neural networks are a reliable method for multi‐omics research because they can effectively manage missing data and noise [67]. In multi‐omics analysis, neural networks of different types, including as feedforward neural networks, RNNs, and CNNs, are employed. Each type of networks is designed to handle many types of input and can be optimized for specific purposes such as classification or prediction. Neural networks are a useful technique for multi‐omics analysis because they may uncover intricate patterns and connections within vast datasets. This skill could offer fresh perspectives and advance our knowledge of complex biological processes.

Neural networks are a common tool used in multi‐omics research that have a number of advantages over traditional statistical methods (Table 2). One major advantage is their ability to do non‐linear mapping of input data to output predictions. This enables neural networks to capture more complex correlations between the input data and the anticipated output in addition to assisting in the discovery of new patterns and insights within multi‐omics datasets. Another advantage of neural networks is their capacity to handle missing data and noise. This is particularly important in multi‐omics research because omics datasets are usually imprecise and noisy. Neural networks can be trained to provide better predictions and analyses by handling missing data and noise [73]. In addition to being used for supervised learning, neural networks may also be used for unsupervised learning, which enables the discovery of patterns and relationships in multi‐omics data without the use of pre‐established labels or objectives. This can be very helpful when creating hypotheses and conducting exploratory data analysis.

**TABLE 2:** Advantages of AI for multi‐omics analysis.

Advantage	Description	References
Automated feature selection	AI algorithms can automatically select features (e.g., genes, proteins, and metabolites) that are most relevant to the biological process of interest, reducing the risk of bias or human error	[68]
Improved accuracy	AI algorithms can process and integrate large amounts of data with high accuracy, detecting patterns and relationships that may not be apparent to human analysts	[69]
Predictive modeling	AI algorithms can build predictive models that can identify disease biomarkers, drug targets, and treatment response predictors from multi‐omics data, aiding in personalized medicine	[70]
Data integration	AI algorithms can integrate multiple types of omics data from different sources and platforms, allowing for a more comprehensive understanding of biological systems	[25]
Time efficiency	AI algorithms can analyze vast amounts of data quickly, saving time and resources compared to traditional data analysis methods	[71]
Scalability	AI algorithms can handle large and complex data sets, making it possible to analyze multi‐omics data at a scale that would be difficult or impossible for humans to manage	[72]

Abbreviation: AI, artificial intelligence.

In multi‐omics analysis, neural networks come in a variety of forms, each with distinct advantages and disadvantages [74]. RNNs are utilized for time‐series data and sequential modeling, while feedforward neural networks are frequently employed for classification and prediction applications. As a formidable tool for multi‐omics analysis, neural networks have the potential to fundamentally alter our understanding of how biological systems work by learning intricate patterns and correlations from vast, heterogeneous datasets.

## 4 APPLICATIONS OF AI AND DATA PROCESSING IN MULTI‐OMICS ANALYSIS

AI and advanced data processing methodologies are fundamentally reshaping the landscape of multi‐omics analysis by effectively addressing various challenges, including data integration, dimensionality reduction, and the intricate interpretation of complex datasets. These applications can be systematically categorized into three principal medical contexts: biomarker identification, disease classification, and therapeutic development. In the realm of biomarker identification, AI methodologies, such as machine learning and neural networks, play a pivotal role in discerning molecular signatures across genomic, transcriptomic, proteomic, and metabolomic datasets, thereby facilitating the early diagnosis and prognosis of diseases [75]. For instance, AI models, including support vector machines and random forests, have been employed to identify biomarkers for cancer subtyping, markedly enhancing diagnostic precision [76, 77]. In the context of disease classification, deep learning frameworks, encompassing CNNs and RNNs, are utilized to stratify patients based on their multi‐omics profiles, thereby supporting precision medicine initiatives.

Through the integration of heterogeneous data sources, these models elucidate disease subtypes and progression dynamics, offering novel insights into personalized therapeutic strategies. This systematic definition highlights the growing importance of AI in the transformation of healthcare. The detection of biomarkers is a crucial use of AI and data processing in multi‐omics study. Biomarkers are quantifiable signs that can be used to diagnose or monitor the development of a disease, evaluate the effectiveness of a treatment, or forecast how a patient will respond to a certain course of action. Through the utilization of AI and data processing methodologies, scholars have the capacity to identify biomarkers associated with specific ailments or maladies by evaluating multi‐omics data [78]. For instance, in cancer research, researchers can analyze genomic, transcriptomic, and proteomic data using AI and data processing to find biomarkers linked to a certain form of cancer. This data can be utilized to create diagnostic tests that can find cancer as well as to create individualized treatment regimens that are catered to each patient’s unique biomarker profile.

Similar to this, in the field of personalized medicine, AI and data processing can be used to evaluate multi‐omics data and find biomarkers linked to a patient’s reaction to a specific medication. This data can be utilized to create individualized treatment plans that are catered to the needs of the patient specifically, leading to more successful and efficient treatment outcomes. The creation of tailored medicine methods is an essential use of AI and data processing in multi‐omics study [79]. Instead of employing a one‐size‐fits‐all strategy, personalized medicine entails adjusting medical care to a patient’s unique genetic, environmental, and lifestyle characteristics. A patient’s distinctive traits can be learned a great deal about through multi‐omics analysis, AI, and data processing, which can then be used to create individualized treatment regimens. Multi‐omics analysis, for instance, can be used to find genetic mutations or other biomarkers linked to a certain disease or condition [80]. With the help of this knowledge, customized medicines can be created that are catered to the individual needs of the patient, leading to improved treatment outcomes and fewer side effects.

Multi‐omics analysis, aided by AI and data processing, provides a powerful means to comprehend the complexities of biological systems, enabling comprehensive understanding of a patient’s unique traits. By amalgamating diverse molecular data types, such as genomics, transcriptomics, proteomics, and meta‐bolomics, multi‐omics analysis offers a comprehensive view of the patient’s molecular profile. In handling and interpreting the vast and intricate data generated from multi‐omics analysis, AI‐driven data processing techniques play a vital role. Machine learning algorithms can identify hidden patterns and correlations within multi‐omics datasets, leading to the identification of potential biomarkers and therapeutic targets [81]. Deep learning models, with their capacity to learn hierarchical representations, can extract meaningful features and relationships from complex omics layers, enhancing the understanding of underlying biological processes. Neural networks enable flexible data computation, allowing for sophisticated integration and analysis of multi‐omics data, which is crucial for advancing personalized medicine strategies [82, 83]. Although the specific AI‐based data processing methods may vary depending on the domain and dataset characteristics, the synergy between AI and multi‐omics analysis holds immense promise in revolutionizing healthcare by providing deeper insights into patients’ individual characteristics and supporting targeted and effective treatment approaches.

AI and data processing can be used to examine massive volumes of data from various sources, including genomic data, imaging data, and electronic health records, to find patterns and associations that would be hard or impossible to find with conventional techniques [84]. With the use of this data, more precise diagnostic procedures and patient‐specific treatment regimens can be created. A better patient experience and more effective use of resources are both possible results of the development of personalized medical methods combining AI and data processing in multi‐omics study.

Our understanding of the mechanisms behind disease can also be advanced with the aid of AI and data processing. AI can discover previously unidentified connections and patterns that may be suggestive of underlying biological pathways underlying disease by evaluating vast and complicated multi‐omics data sets [85]. As a result, new theories and insights into the etio‐logy and course of diseases may be developed by researchers, which may ultimately result in the creation of novel therapies. For instance, AI has been applied to the analysis of multi‐omics data sets in cancer research, revealing novel biomarkers and previously undiscovered potential therapeutic targets [86, 87]. AI can give a more complete picture of the illness and pinpoint important pathways and mechanisms that may be involved in the onset and progression of cancer by merging data from the fields of genomes, transcriptomics, proteomics, and metabolomics. This knowledge can aid in the development of more potent targeted treatments for cancer patients [86].

AI has been used to assess multi‐omics data in various disease domains, such as cardiovascular and neurodegenerative disorders [88]. AI can assist in better comprehending the underlying causes of various diseases and creating more efficient treatments by finding important molecular pathways and biomarkers [89]. AI can also assist in finding prospective pharmacological targets and repurposing already existing medications for novel therapeutic applications. AI can locate important proteins and disease‐related pathways by analyzing multi‐omics data sets, and it can then search through databases to find medications that target these proteins. The process of finding new drugs and developing them can be greatly sped up and less expensive. Our understanding of disease causes, and the creation of novel medicines could be completely changed using AI and data processing in multi‐omics study.

There is study to understand how cells coordinate gene expression in response to environmental stimuli, specifically in resting and stimulated human blood cells [90]. Used single‐cell multi‐omics techniques, including scATAC‐seq and scRNA‐seq, to generate over 91,000 single‐cell profiles and probe the *cis*‐regulatory landscape of the immunological response across cell types, stimuli, and time. They developed a framework called functional inference of gene regulation to computationally pair scATAC‐seq with scRNA‐seq cells, connect distal *cis*‐regulatory elements to genes, and infer gene‐regulatory networks to identify candidate TF regu‐lators. The goal of this research is to elucidate regulatory interactions across single‐cell data and provide new opportunities to understand the function of cells within tissues.

## 5 CHALLENGES AND FUTURE PERSPECTIVES

One of the primary obstacles encountered in multi‐omics research is the integration and preprocessing of data. Advanced techniques are required to address the challenges of integrating diverse and heterogeneous datasets from different sources and experimental platforms, including handling variations, harmonizing data, and effectively addressing missing values. Additionally, the complexity and high dimensionality of data pose significant challenges, leading to overfitting and computational burdens. In employing deep learning models, interpretability and explainability are critical aspects that must be considered. The inherent complexity of deep learning algorithms makes it challenging to interpret learned representations and understand the underlying biological mechanisms driving predictions [91]. The issue of sample size limitations must also be addressed to ensure the generalization capability of AI models.

Collaboration between biologists, clinicians, data scientists, and statisticians can foster an interdisciplinary understanding and address the unique challenges at the intersection of AI and multi‐omics research. Continued research and innovation in advanced algorithms and models specifically designed for multi‐omics data ana‐lysis can enhance the performance and interpretability of AI models. Developing novel techniques that enable transfer learning and data augmentation can leverage knowledge from related tasks and synthetic data to improve model generalization and deal with limited samples effectively. Interpretability techniques are crucial for understanding the underlying biological context of AI predictions [92].

New methodologies are needed to unravel the complex decisions made by deep learning models and provide meaningful insights to domain experts. Encouraging data sharing and standardization is crucial to enhance the reproducibility and reliability of multi‐omics studies. Adopting standardized protocols for data acquisition, preprocessing, and analysis can facilitate the integration of diverse datasets from different sources. Moreover, ethical considerations play a vital role in the success of multi‐omics research using AI [93]. Establishing ethical guidelines for handling sensitive data and ensuring data privacy and security are imperative for maintaining public trust and responsible use of AI in biomedical research.

Researchers have access to a plethora of platforms, libraries, and frameworks that enable them to perform diverse analysis tasks, from statistical analyses to deep learning‐based modeling (Table 3). These open‐source tools provide not only a cost‐effective solution but also offer the flexibility to customize and extend functionalities according to specific research needs. The domain of multi‐omics analysis harbors enormous potential for future advancements, particularly in the realm of advanced AI algorithms. With the evolution of machine and deep learning techniques, there is a rising demand for algorithms that can tackle the complexities of multi‐omics data. Future research endeavors will be dedicated to refining current algorithms and devising innovative approaches aimed at addressing challenges such as data heterogeneity, high dimensionality, and small sample sizes.

**TABLE 3:** Open‐source tools play a crucial role in revolutionizing multi‐omics analysis with AI and data processing.

Open‐source tool	Description	Potential applications
MultiQC	A tool designed to aggregate and visualize results from bioinformatics analyses, often used in multi‐omics workflows to combine and interpret outputs from various omics platforms	Quality control for multi‐omics pipelines
mixOmics	Provides methods for the integrative analysis of multi‐omics data, offering dimension reduction techniques tailored for correlation and interaction studies	Canonical correlation analysis
Galaxy	An open‐source platform for multi‐omics data integration and analysis with extensive toolsets supporting diverse omics data workflows	Cross‐platform omics data integration
Expasy	A bioinformatics resource portal for multi‐omics analysis, including protein and metabolomics integration	Data annotation

Abbreviation: AI, artificial intelligence.

The integration of AI in multi‐omics analysis will witness substantial progress in the realm of interpretability and explainable AI. As AI models become more intricate, comprehending the rationale behind their predictions becomes critical for gaining trust and acceptance in biomedical research and clinical applications [94]. Future research will focus on developing interpretable AI models that can provide transparent insights into the underlying biological mechanisms driving the predictions. Explainable AI will not only enhance the credibility of AI‐driven findings but also facilitate knowledge discovery and hypothesis generation by allowing domain experts to comprehend and validate the model’s decisions.

The integration of epigenomic data with genomic and transcriptomic datasets has proven to be pivotal in the identification of epigenetic biomarkers pertinent to cancer prognosis, exemplified by hypermethylated CpG islands located within tumor suppressor genes [95]. The application of sophisticated machine learning methodologies, including dimensionality reduction and feature selection algorithms, is increasingly prevalent in the analysis of high‐dimensional epigenomic data, facilitating the identification of regulatory elements and their interactions with other omics layers. Epigenomics provides vital insights when synergistically combined with other omics data to decode disease progression and therapeutic resistance [96]. In the domain of precision medicine, the amalgamation of methylation profiles with transcriptomic data has unveiled epigenetically regulated gene expression networks that are predictive of patient responses to targeted therapeutic interventions. Likewise, the integration of chromatin accessibility data with proteomics has facilitated the identification of TF binding motifs, thereby illuminating regulatory pathways.

One of the most promising future perspectives in multi‐omics analysis lies in advancing integrative techniques. Research endeavors will be geared towards devising methodologies that can seamlessly combine genomics, transcriptomics, proteomics, metabolomics, and other omics data. Integrative multi‐omics analysis will open up new avenues for identifying biomarkers, understanding disease mechanisms, and designing personalized treatment strategies [79, 97]. The burgeoning field of single‐cell multi‐omics holds immense potential for revolutionizing our understanding of cellular heterogeneity and dynamics. As technology continues to advance, single‐cell multi‐omics data will become more accessible, leading to new insights into cellular states and interactions. Future research will center around the development of specialized AI methods to analyze and interpret single‐cell multi‐omics data. Leveraging the power of AI in this context will unlock invaluable knowledge about cellular processes, developmental pathways, and disease progression at unprecedented resolution.

Collaboration between researchers, institutions, and industry stakeholders will be instrumental in driving multi‐omics research forward. The future of multi‐omics analysis will witness increased collaboration to tackle complex challenges collectively. Data sharing initiatives will facilitate the creation of large, diverse datasets that can be leveraged to validate findings and generalize discoveries across different populations and diseases. By fostering a collaborative environment, the scientific community can accelerate the pace of multi‐omics research and enhance the reproducibility and reliability of AI‐driven analyses. As AI models become more refined, their capacity to predict disease risk, identify patient subgroups, and recommend personalized therapies will be greatly enhanced. Future research will focus on the integration of multi‐omics data into clinical practice, enabling clinicians to make data‐driven decisions tailored to each patient’s unique molecular profile. Precision medicine powered by AI‐driven multi‐omics analysis has the potential to revolutionize healthcare, leading to better patient outcomes and more efficient healthcare delivery.

## 6 CONCLUSION

Multi‐omics analysis could be revolutionized, and data processing techniques and AI could change how we perceive complicated biological systems. For the development of customized medical strategies, the discovery of novel biomarkers, and the advancement of our understanding of disease mechanisms, the capacity to evaluate and integrate huge and complex data sets is essential. However, there are also significant difficulties in using data processing and AI tools for multi‐omics analysis. These include the requirement for good data sets, suitable models and algorithms, and the creation of standardized methods for data processing and analysis. It is crucial to build collaborative and multidisciplinary research efforts in order to fully utilize AI and data processing in multi‐omics analysis. Experts in biology, computer science, and statistics will need to be involved, and solid, open‐source procedures for data analysis and interpretation will need to be developed. In conclusion, the fusion of data processing techniques and AI has the potential to revolutionize multi‐omics analysis and advance the creation of personalized medical strategies. But it would take more research and collaboration to find ways to use these technologies effectively.

## AUTHOR CONTRIBUTIONS

**Ali Yetgin**: Conceptualization; visualization; writing—original draft; writing—review and editing.

## CONFLICT OF INTEREST STATEMENT

The author declares no conflicts of interest.

## ETHICS STATEMENT

This review article is based on a comprehensive ana‐lysis of existing literature and does not involve human or animal subjects, experimental research, or personal data collection.

## Data Availability

No new data were created or analyzed in this study. Data sharing is not applicable to this article.
